# PReS-FINAL-2352: Apoptosis profile in patients with juvenile-onset systemic lupus erythematosus

**DOI:** 10.1186/1546-0096-11-S2-P342

**Published:** 2013-12-05

**Authors:** B Liphaus, MH Kiss, S Carrasco, C Goldenstein-Schainberg, Faculdade de Medicina da Universidade de São Paulo, São Paulo, Brazil

**Affiliations:** 1 Reumatologia, Faculdade de Medicina, Universidade de Sao Paulo, Sao Paulo, Brazil

## Introduction

Apoptosis related proteins have been involved in immune dysregulation and development of systemic lupus erythematosus (SLE).

## Objectives

To assess sFas, sFasL, sTRAIL and sBcl-2 in sera and to evaluate Fas and Bcl-2 expressions in peripheral monocytes, T and B lymphocytes from juvenile-onset SLE (JSLE) and to determine relationships with disease activity.

## Methods

Forty-three JSLE patients (revised ACR criteria, mean age = 14.3 yrs, 36F:7M), and 35 age and gender matched healthy controls were studied; 30 JSLE had SLEDAI score^3 ^4, reflected active disease. Soluble molecules were measured by commercial ELISA kits. Lymphocytes and monocytes were stained with specific moAbs and analyzed by flow cytometry. Kruskal-Wallis test and Spearman's rank were employed and statistical significance considered p value < 0.05.

## Results

JSLE sera had significantly increased sFas (188.1 ± 69.2 vs 133.2 ± 80.6, pg/ml) and sTRAIL (691.3 ± 631.8 vs 346.6 ± 251.1, pg/ml), decreased sFasL (0.08 ± 0.1 vs 0.36 ± 0.4, ng/ml), and similar sBcl-2 (7.4 ± 8.6 vs 9.3 ± 9.6, mg/ml) levels compared to healthy controls. SLEDAI score directly correlated with sFas (r = 0.52; p = 0.001). JSLE patients compared to controls had significantly increased Fas expression on CD3+ (43.7 ± 10.3% vs 28.9 ± 9.4%), CD4+ (20.3 ± 6.7% vs 16.2 ± 6.2%) and CD8+ (21.5 ± 9.6% vs 12.3 ± 5.8%) T cells, and also on CD19+ B cells (2.1 ± 1.4% vs 1.4 ± 0.7%), whereas, it was decreased on CD14+ monocytes (93.6 ± 6.9% vs 96.7 ± 2.5%, p = 0.01). There was direct correlation between percentages of CD19+Fas+ cells and SLEDAI (r = 0.38, p = 0.02) and inverse correlation between percentages of CD14+Fas+ cells and SLEDAI (r= -0.55, p = 0.01). Mean fluorescence intensity (MFI) of Bcl-2-positive cells from JSLE patients was significantly increased in CD3+ (28.8 ± 8.4 vs 22.9 ± 4.2), CD4+ (28.6 ± 8.2 vs 22.9 ± 4.4) and CD8+ (29.4 ± 9.4 vs 22.8 ± 3.6) T cells, and also in CD19+ B cells (25.5 ± 9.6 vs 21.5 ± 3.6). Bcl-2 expression in CD14+ monocytes was lower in JSLE compared to controls (25.2 ± 18.2% vs 34.5 ± 16.6%, p = 0.006). Direct correlation between percentages of CD19+Bcl-2+ cells and SLEDAI (r = 0.47, p = 0.04) was shown.

## Conclusion

JSLE patients showing high sFas and sTRAIL and low sFasL levels with Fas and Bcl-2 expressions increased on circulating T and B lymphocytes though decreased on monocytes are remarkable evidences of apoptosis role in the immune dysregulation observed. A possible role as a marker for lupus disease activity needs to be defined.

## Disclosure of interest

None declared.

